# A Review of the Phenotype of Synpolydactyly Type 1 in Homozygous Patients: Defining the Relatively Long and Medially Deviated Big Toe with/without Cupping of the Forefoot as a Pathognomonic Feature in the Phenotype

**DOI:** 10.1155/2020/2067186

**Published:** 2020-05-15

**Authors:** Mohammad M. Al-Qattan

**Affiliations:** Division of Plastic and Hand Surgery, Department of Surgery at King Saud University, Riyadh, Saudi Arabia

## Abstract

Synpolydactyly type 1 (SPD1, OMIM 186000) is inherited as autosomal dominant and is caused by *HOXD13* mutations. The condition is rare and is known for its phenotypic heterogeneity. In the homozygous state, the phenotype is generally more severe and is characterized by three main features: a more severe degree of syndactyly, a more severe degree of brachydactyly, and the frequent loss of the normal tubular shape of the metacarpals/metatarsals. Due to the phenotypic heterogeneity and the phenotypic overlap with other types of syndactyly, no pathognomonic feature has been described for the homozygous phenotype of SPD1. In the current communication, the author reviews the literature on the phenotypes of SPD1 in homozygous patients. The review documents that not all homozygous patients show a severe hand phenotype. The review also defines the “relatively long and medially deviated big toe with/without cupping of the forefoot” as a pathognomonic feature in the phenotype. Illustration of this feature is done through a demonstrative clinical report in a multigeneration family with SPD1 and *HOXD13* polyalanine repeat expansion. Finally, the pathogenesis of the clinical features is reviewed.

## 1. Introduction

Synpolydactyly type 1 (SPD1) is inherited as autosomal dominant and is caused by *HOXD13* mutations. It has also been given other names such as syndactyly type IIA and the Vordingborg syndactyly [1]. In the heterozygous state, classic hand features include fusion of the third and fourth fingers with duplication within the syndactylous web. In the feet, there is classically cutaneous webbing of the fourth and fifth toes. In the homozygous state, the phenotype is generally more severe and this was reviewed by Malik and Grzeschik [2] and Al-Qattan [3]. Malik and Grzeschik [2] stressed on the extreme phenotypic heterogeneity in SPD1 and classified the clinical variants into three categories according to the degree of severity of the phenotype: heterozygous patients showing a very mild phenotype (frequently, clinodactyly or camptodactyly of the little finger is the only manifestation of the gene mutation), patients with classic SPD1 features (usually seen in heterozygous patients but may be seen in homozygous patients), and homozygous patients with severe phenotypes. Al-Qattan [3] stressed on the three main features of the homozygous phenotype: syndactyly frequently involves the postaxial three or four digits, a more severe degree of concurrent brachydactyly, and the frequent loss of the normal tubular shapes of the metacarpals/metatarsals (they may become polygonal in shape; and in some cases, they attain the shape of the carpal/tarsal bones). However, these severe features are not always seen in every homozygous patient. Due to the phenotypic heterogeneity and the phenotypic overlap with other types of syndactyly, no pathognomonic feature has been described for the homozygous phenotype of SPD1.

In the current communication, the author reviews the literature on the phenotype of SPD1 in homozygous patients and defines the “relatively long and medially deviated big toe with/without cupping of the forefoot” as a pathognomonic feature of the phenotype. Illustration of this feature is done by a demonstrative clinical report. Finally, the pathogenesis of the clinical features is reviewed.

### 1.1. A Review of the Different *HOXD13* Mutations Associated with SPD1

The *HOXD13* gene codes for a protein with 343 amino acids. The protein normally carries a 15-amino acid polyalanine repeat in the N-terminus, while the DNA-binding homeodomain is at the C-terminus [4]. Three types of *HOXD13* mutations have been associated with SPD1: expansions of the N-terminal polyalanine repeat, missense mutations at either the N- or C-terminus, and putative null or loss-of-function mutations (such as the nonsense mutations) [4]. Alterations in function with these mutations have also been studied. Polyalanine repeat expansions result in cytoplasmic aggregation of the mutant HOXD13 protein [5]. The degree of aggregation is influenced by the length of the repeat, and hence, there is a correlation between the *HOXD13* expansion size and the severity of the phenotype [6]. Missense mutations at the N-terminus result in a reduction in the half-life of the mutant HOXD13 protein; and experimentally, there is interference with Gli3R function during limb prepatterning [7]. Missense mutations at the C-terminus prevent binding to the DNA-binding domain [4]. Finally, truncating mutations result in loss-of-function.

### 1.2. A Review of Previously Reported Families with SPD1 and Homozygous Patients

Tables 1 and 2 summarize the phenotype of all previously reported families with homozygous patients. There were four families with polyalanine repeat expansions [8–12]. The phenotype in the heterozygous parents varied from a very mild phenotype (such as isolated clinodactyly of the little finger) to the classic phenotype described in the introduction. Homozygous patients had a more severe syndactyly involving the postaxial 3 fingers/toes, a variable degree of brachydactyly, and the loss of the normal tubular shape of metacarpals/metatarsals. Brachydactyly was more pronounced in the postaxial digits and mostly affected the middle phalanges; and in some cases, the middle phalanx was absent. In all homozygous patients, the big toe was relatively long and medially deviated. Cupping of the forefoot (leading to plantar flexion of the postaxial toes) was not seen in all homozygous patients. It is important to note that the big toe/forefoot feature was not specifically mentioned by the authors; but the feature was clear in the illustrations.

There were two families with missense mutations [4, 7] and one family with a truncating nonsense mutation [13]. The phenotypes in these 3 families are summarized in Table 2. All heterozygous parents were either normal (i.e., with nonpenetrance) or with a very mild phenotype (such as isolated camptodactyly of the little finger). The phenotype in homozygous patients was more severe, and all patients had the big toe/forefoot cupping feature. Once again, this pathognomonic feature was noted by the current author from the illustrations; and the feature was not specifically mentioned by the authors of these reports. The phenotype in the homozygous patient described by Ibrahim et al. [4] illustrated the significant variation of the well-known phenotypic characteristics of homozygous patients. Hand syndactyly was mild in that homozygous patient (one hand had no syndactyly, and the other hand had syndactyly of the 3rd web without polydactyly). In contrast, the degree of loss of the normal shape of the metacarpals in the same patient was severe; and metacarpals attained the shape of carpal bones [4]. This demonstrated that the three characteristic features of the homozygous phenotype (as described by [3]) may not all be present in every patient. In fact, all three features may be lacking in homozygous patients. For example, syndactyly, brachydactyly, and shape changes of the metacarpals/metatarsals were all mild in the homozygous patients reported by Brison et al. [7].

### 1.3. A Demonstrative Clinical Report

**Figure 1:**
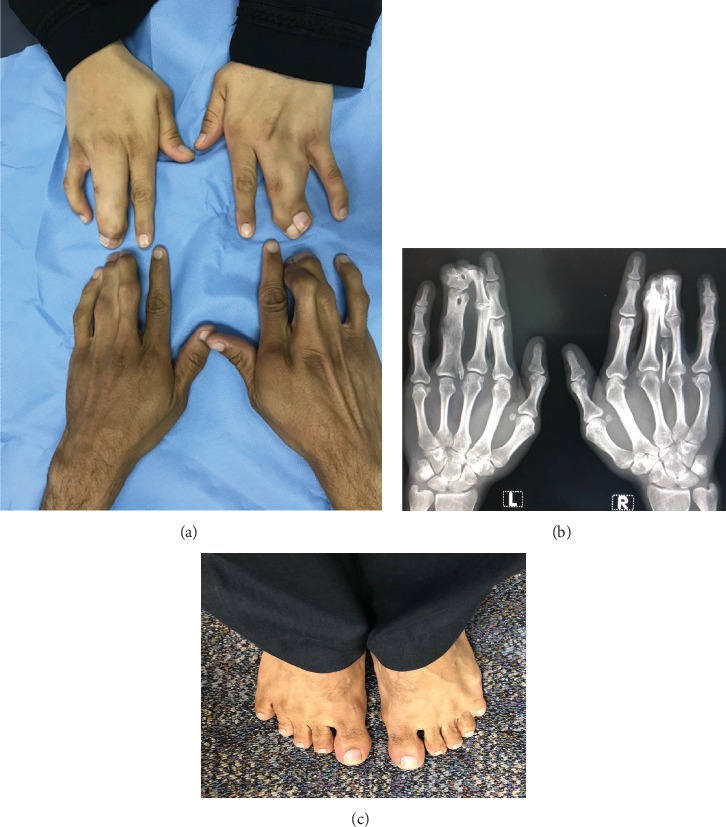
The phenotypes in the heterozygous parents: (a) the hands of the mother (above) and father (below) showing synpolydactyly of the third and fourth digits; (b) X-ray of the hands of the father showing the duplication within syndactyly; (c) the feet of the father showing bilateral little toe brachydactyly and webbing between the 4th and 5th toes in the left foot.

**Figure 2:**
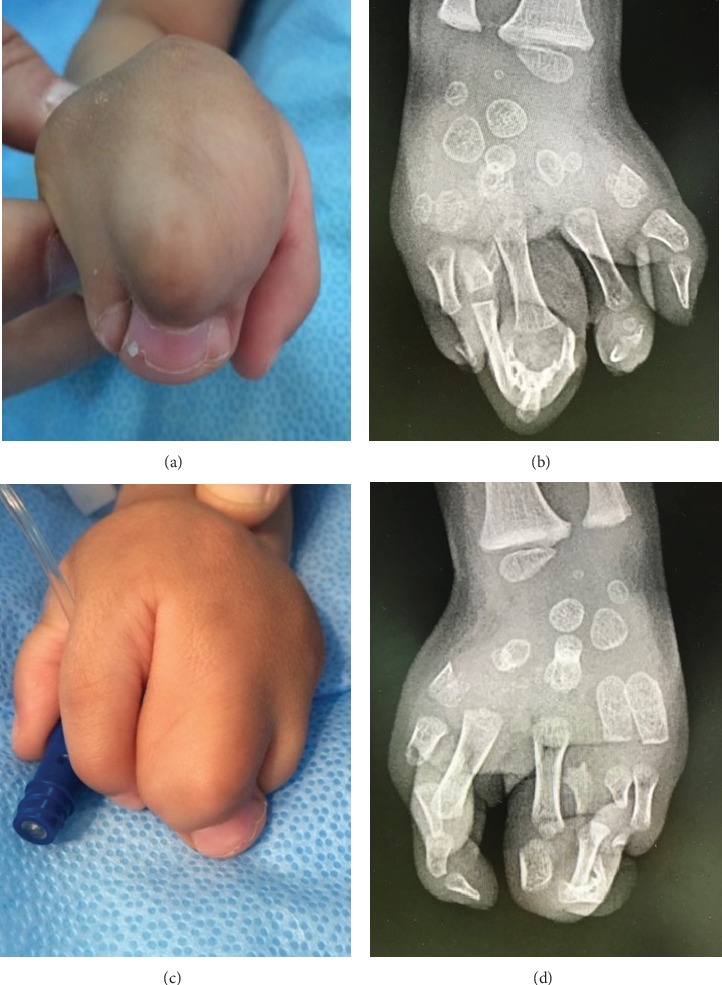
The hands of the homozygous child: (a) clinical appearance of the right hand showing syndactyly of all fingers and “cupping” of the hand; (b) X-ray of the right hand (after surgical separation of the index finger). Note that the metacarpals have attained the shape of carpal bones; (c) clinical appearance of the left hand showing syndactyly of the middle, ring, and little fingers; (d) X-ray of the left hand also showing a metacarpal-to-carpal transformation.

**Figure 3:**
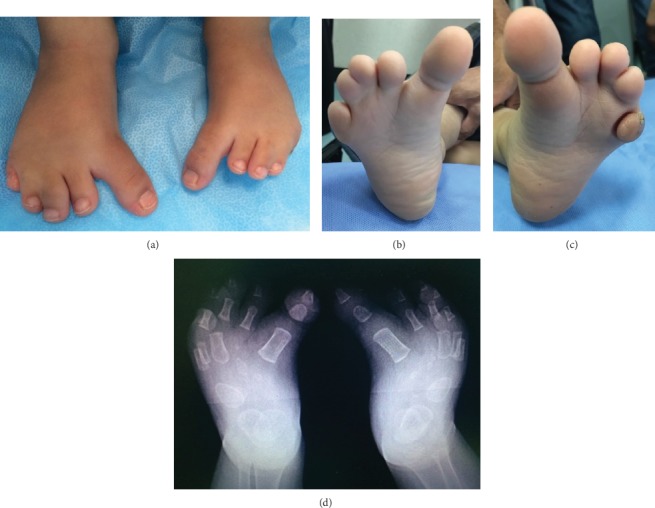
The feet of the homozygous child: (a) note the relatively long and medially deviated big toes. Also, note the plantar flexion deformity of the postaxial four toes; (b) cupping of the forefoot on the right; (c) cupping of the forefoot on the left; (d) X-rays of the feet. Note the metatarsal-to-tarsal transformation.

The family of the index patient is a multigeneration family with features of SPD1. The parents are affected first cousins with classic features of SPD1 (Table 3, Figure 1). The parents never had surgical correction of their deformities. Their boy had a severe phenotype, and the parents presented the child to the author requesting surgical correction of his deformities. The pregnancy was uneventful. Anthropometric measurements revealed normal stature, weight, and head circumference. Systemic examination showed no abnormalities, and ultrasound of the abdomen was normal. All abnormalities were confirmed to the hands and feet. His phenotype is summarized in Table 3 and Figures 2 and 3. Relatively long and medially deviated big toes and cupping of forefeet (leading to plantar flexion deformities of the lateral four toes) were noted bilaterally (Figure 3).

Venous blood samples were obtained from the parents and child after a written informed consent. Whole-exome sequencing (CentoXome GOLD®) was performed. The index case was found to be homozygous and the parents were found to be heterozygous for the following variant in the *HOXD13* gene: c.209_210insGGCTGCGGCGGCGGCAGCGGC p.(Ala65_Ala71dup) which is an in-frame insertion of 21 bps in exon 1, which causes the duplication of 7 residues. The variant has been confirmed by Sanger sequencing. It is classified as pathogenic (class 1) according to the recommendations of Centogene and ACMG. Kjaer et al. [14] reported this variant as disease-causing for SPD1 in one large family with segregation.

### 1.4. A Review of the Pathogenesis of the Clinical Features of the Homozygous Phenotype

This will be discussed separately for syndactyly. The pathogenesis of brachydactyly, shape changes for the metacarpals/metatarsals, and the big toe pathognomonic feature will be grouped together since these features have the same pathogenesis.

### 1.5. How Does a Defective HOXD13 Protein Cause Syndactyly?

The author has previously offered a 3-step unified pathway of pathogenesis for syndactyly [15]. In the first step, there is either the overactivation of the WNT canonical pathway or the suppression of the bone morphogenetic protein canonical pathway. This leads to an overexpression of fibroblast growth factor 8, which is considered the second step. The final and third step is the suppression of retinoic acid in the interdigital mesenchyme leading to suppression of both apoptosis and extracellular matrix degradation and hence resulting in syndactyly. A defective HOXD13 acts on the third step because experimental models have shown that the mutated *Hoxd13* has a direct suppressive effect on retinoic acid in the autopod [16]. Hence, the homozygous phenotype is expected to have a more severe syndactyly.

### 1.6. The Pathogenesis of Brachydactyly, Shape Changes for the Metacarpals/Metatarsals, and the Big Toe Pathognomonic Feature

The expression of HOXD13 in the digital zones during development has a major influence on the length of bones within the digital rays [17]. As the expression of HOXD13 decreases, the degree of brachydactyly increases. Hence, brachydactyly in homozygous patients is expected to be more severe than in heterozygous patients. Other heterozygous mutations of *HOXD13* cause isolated brachydactyly such as brachydactyly type E (OMIM113300), and the reason for this is unclear.

The big toe is the preaxial digit in the foot; and our review shows that there is relative preservation of the length of the big toes in the homozygous SPD1 phenotype. It is important to note that the length of the big toe is normal, but it appears relatively long compared to the brachydactylous postaxial toes. This may be explained by the well-known distribution of HOXD13 activities within the autopod. HOXD13 has the lowest expression in the preaxial digit compared to other digits [18]. In contrast, HOXA13 is highly expressed in the preaxial digit and influences its development and length. In mice, Hoxa13 loss of function results in the lack of formation of all preaxial digits [19]. In humans, mutations in *HOXA13* cause the hand-foot-uterus syndrome (OMIM 140000) which typically presents with short thumbs and big toes [20]. Hence, the degree of brachydactyly associated with a defective HOXD13 protein is expected to be less pronounced in the preaxial digit because of an undisturbed HOXA13.

The mesopod (the area of carpal bones) normally has no HOXD13 expression, and this zone is sometimes called the “no HOXD land” [18]. This brought up the theory that the length of the bones in any zone within the autopod will decrease and will eventually attain the shape of carpal bones as the HOXD13 activity is decreased [18]. Hence, the severe loss of HOXD13 activity in the zones of metacarpals/metatarsals may explain the metacarpal/metatarsal-to-carpal/tarsal transformation seen in some homozygous patients with SPD1 (see Figures 2(b) and 2(d) and 3).

## 2. Conclusions

The current report is the most comprehensive review of the phenotypes of SPD1 in homozygous patients. The review documents that not all homozygous patients show a severe hand phenotype. The review also defines the “relatively long and medially deviated big toe with/without cupping of the forefoot” as a pathognomonic feature in the phenotype.

## Figures and Tables

**Table 1:** A summary of the phenotypes in the heterozygous parents and their homozygous children in previously reported families with SPD1 caused by *HOXD13* polyalanine repeat expansions.

		The phenotype in their homozygous children is divided into five categories			
Authors	The phenotype in the heterozygous parents	1—syndactyly	2—brachydactyly	3—the shapes of metacarpals/metatarsals	4—the big toe sign	5—cupping of the forefeet
Akarsu et al. [8]; Akarsu et al. [9]	Mild or classic SPD1 phenotype in the hands and feet. Some parents had no abnormalities (i.e., nonpenetrant)	Involved the postaxial 3-4 digits in the hands and feet	Moderate to severe in the hands, mild to moderate in the feet	Short broad metacarpals	Yes	Yes

Muragaki et al. [12]	Classic phenotypes in the hands and feet	Involved the postaxial 3 digits in the hands	Moderate and more pronounced in the middle phalanges of the fingers and toes	Polygonal metacarpals, short broad second metatarsals, replacement of metatarsals III-IV with a single tarsal-like bone	Yes	No

Horsnell et al. [10]	Classic phenotypes in the hands and feet	Involved the postaxial 3 digits in the hands and feet	Moderate and more pronounced in the middle phalanges of the fingers and toes	Broad or polygonal metacarpals. The metatarsals were relatively preserved	Yes	No

3 et al. (2007)	Classic or mild phenotype. Some had a mild phenotype (with normal feet). Partial duplication of the first metatarsal was also seen	Involved the postaxial 3 digits in the hands and feet	Moderate and more pronounced in the middle phalanges of the fingers and toes	Broad or polygonal metacarpals. The metatarsals were relatively preserved	Yes	No

**Table 2:** A summary of the phenotypes in the heterozygous parents and their homozygous children in previously reported families with SPD1 caused by *HOXD13* missense and truncating mutations.

		The phenotype in their homozygous children is divided into five categories			
Mutations in *HOXD13* (authors)	The heterozygous parents	1—syndactyly	2—brachydactyly	3—the shapes of metacarpals/metatarsals	4—the big toe sign	5—cupping of the forefeet
G11A (missense at the N-terminus) [7]	Very mild phenotype: bilateral little finger camptodactyly, brachydactyly of the 4^th^ and 5^th^ toes	Classic SPD1 (digits 3-4 in the hands and digits 4-5 in the feet)	Mild in the hands and feet, more pronounced in the middle phalanges	Some metacarpals were slightly broad. The metatarsals were preserved	Yes	Yes

T313R (missense at the DNA-binding domain) [4]	No abnormalities (i.e., nonpenetrance)	One hand had no syndactyly; the other hand had digits 3-4 syndactyly. Both feet had cutaneous syndactyly of the middle 3 toes	Moderate in the hands and feet, more pronounced in the middle phalanges	Metacarpal-to-carpal transformation. The metatarsals were broad or polygonal	Yes	Yes

Q248X (nonsense) [14]	Very mild phenotype (isolated little finger clinodactyly) or classic SPD1 phenotype	Usually involved the middle, ring, and little fingers in the hands. Variable in the feet but frequently involved the postaxial 4 toes	Moderate in the hands and mild in the feet	Preserved	Yes	Yes

**Table 3:** A summary of the phenotypes in the heterozygous parents and their homozygous child of the family reported in the current review.

	The heterozygous mother	The heterozygous father	The homozygous child
The hands	Classic SPD1 hand phenotype: isolated synpolydactyly of the middle and ring fingers	Classic SPD1 hand phenotype: isolated synpolydactyly of the middle and ring fingers	Syndactyly involved all fingers in the right hand and the postaxial 3 fingers in the left hand. Cupping of the right hand was noted. There was metacarpal-to-carpal transformation bilaterally.
The feet	No abnormalities	Classic SPD1 foot phenotype: webbing of the 4^th^ and 5^th^ toes in the left foot. Bilateral little toe brachydactyly was also present	The big toes were relatively long and medially deviated. Cupping of the forefeet was also noted bilaterally leading to plantar flexion deformity of the toes. Cutaneous syndactyly was seen between the 3^rd^ and 4^th^ toes bilaterally. X-rays showed metatarsal-to-tarsal transformation bilaterally.
